# A connection between proto-neutron-star Tayler–Spruit dynamos and low-field magnetars

**DOI:** 10.1038/s41550-025-02477-y

**Published:** 2025-02-04

**Authors:** Andrei Igoshev, Paul Barrère, Raphaël Raynaud, Jérome Guilet, Toby Wood, Rainer Hollerbach

**Affiliations:** 1 School of Mathematics, Statistics and Physics, Newcastle University, Newcastle upon Tyne, UK; 2 Department of Applied Mathematics, University of Leeds, Leeds, UK; 3 Observatoire de Genève, Université de Genève, Versoix, Switzerland; 4 Université Paris-Saclay, Université Paris Cité, CEA, CNRS, AIM, Gif-sur-Yvette, France; 5 Université Paris Cité, Université Paris-Saclay, CEA, CNRS, AIM, Gif-sur-Yvette, France

**Keywords:** Compact astrophysical objects, Astrophysical magnetic fields, Stars, High-energy astrophysics

## Abstract

Low-field magnetars have dipolar magnetic fields of 10^12^–10^13^ G, 10–100 times weaker than the values of magnetic-field strength *B* ≈ 10^14^–10^15^ G used to define classical magnetars, yet they produce similar X-ray bursts and outbursts. Using direct numerical simulations of magnetothermal evolution starting from a dynamo-generated magnetic field, we show that the low-field magnetars can be produced as a result of a Tayler–Spruit dynamo inside a proto-neutron star. We find that these simulations naturally explain key characteristics of low-field magnetars: weak (≲10^13^ G) dipolar magnetic fields, strong small-scale fields and magnetically induced crustal failures producing X-ray bursts. These findings suggest that the formation channel of low-*B* magnetars is distinct from that for classical magnetars, reflecting potential differences in proto-neutron-star dynamos.

## Abstract (web summary)

Stellar material falling back in supernova explosions plays a crucial role in the formation of magnetic fields for some magnetars. X-ray bursts of these ‘low-field’ magnetars are products of their complicated fields, produced in Tayler–Spruit dynamos.

## Main

Magnetars play a special role in modern high-energy astrophysics. They have been suggested as central engines for superluminous supernovae^1^ and ultralong γ-ray bursts^2^. They produce at least a fraction of the mysterious fast radio bursts^3,4^. While Galactic magnetars are scarce due to their short life—with 30 known magnetars, compared with 3,500 radio pulsars according to Australia Telescope National Facility Pulsar Catalogue v.2.1.1^5^—it is estimated that around 10% of all neutron stars (NSs) are observed as magnetars at some point in their evolution^6^.

The standard magnetar model explains quiescent X-ray emission, spin period, bursts, outbursts and giant flares observed from anomalous X-ray pulsars and soft gamma repeaters (SGRs) by assuming that these NSs have strong dipolar magnetic fields, approximately 10^14^–10^15^ G (refs. ^7,8^). However, a substantial fraction of magnetars (5 out of 30 known objects) in fact have dipolar magnetic fields well below 10^14^ G and have therefore been named low-field magnetars^9–12^. It has been suggested that low-field magnetars are old NSs primarily powered by crust-confined toroidal magnetic fields with strength of approximately 10^14^ G (refs. ^10,13^). Rea et al.^11^ suggested that low-field magnetars are born with both poloidal and toroidal magnetic fields greater than 10^14^ G, but that the poloidal component decays by a factor of six in approximately 500 kyr. Phase-resolved X-ray observations show that in two cases low-field magnetars host small-scale magnetic fields which are 10–100 times stronger than their dipolar fields^14,15^.

The origin of magnetar magnetic fields is a subject of debate^16^. Different dynamo mechanisms have been proposed to explain the formation of the strongest magnetic fields, including proto-NS convection^7,17–19^, magnetorotational instability (MRI)^20^ and more recently the Tayler–Spruit dynamo^21–23^. The Tayler–Spruit dynamo is a particularly promising mechanism for generating magnetars’ magnetic fields in cases when the progenitor core is slowly rotating and the proto-NS is spun up by fallback accretion^22^. In cases of rotation periods larger than 5 ms, a normal core-collapse supernova is expected to occur, in agreement with observational constraints for the majority of magnetars^24,25^. After the first minute, the proto-NS cools down, it shrinks in radius, crust solidification begins and the remnant becomes an NS. After this time, the initially complicated crustal magnetic field slowly relaxes due to Ohmic decay and Hall evolution on a timescale of 10^5^–10^6^ yr (ref. ^26^).

Previous simulations of magnetothermal evolution have assumed idealized initial conditions (dipole, quadrupole)^13^ or random small-scale fields^27–30^ rather than detailed magnetic configurations generated by a specific dynamo mechanism. However, the study of more realistic initial conditions is of key importance to obtain realistic predictions of magnetar properties. Indeed, Hall evolution has been shown to preserve certain aspects of the initial conditions^31,32^. Hence, the observational properties of magnetars, and low-field magnetars in particular, should contain information about the proto-NS magnetic field.

## Evolution of NS magnetic field

The proto-NS dynamo and NS crust stages are modelled separately because of their very different timescales and physical conditions. While the dynamo is formulated as a magnetohydrodynamics (MHD) problem for a stably stratified fluid with shear caused by fallback accretion (which is mimicked through the boundary condition, see Methods for more details) over a timescale of a few tens of seconds, the magnetothermal evolution of the NS crust occurs on a much longer timescale of 1 Myr and is formulated as electron MHD (eMHD).

The initial condition for our NS simulation is a magnetic-field configuration taken from the late stages of model Ro0.75s from ref. ^23^ (Table 1 in their supplemental materials) and corresponds to a Tayler–Spruit dynamo branch recently discovered in direct numerical simulations and characterized by a dipolar symmetry (that is, equatorially symmetric)^23^. The radius of the proto-NS at this stage is 12 km, which is very similar to the NS radius. This magnetic field is obtained using the three-dimensional spherical MHD code MagIC^33^ for rotation frequencies of the outer and inner spheres respectively *Ω*_o_ = 4*Ω*_i_ = 628 rad s^−1^ (which corresponds to a surface rotation period of 10 ms; see Methods for a more detailed description).

**Extended Data Fig. 1:**
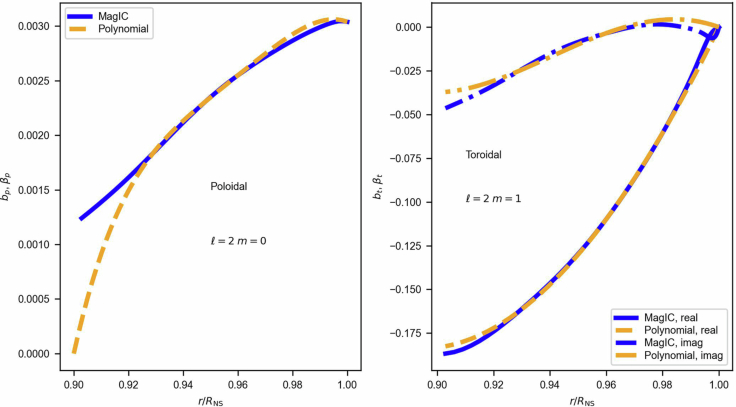
Radial function extracted from the MHD simulations compared with its polynomial model. Left panel: poloidal potential for *ℓ*=2, *m*=0. Right panel: toroidal potential for *ℓ*=2, *m*=1 which has both real and imaginary parts.

**Extended Data Fig. 2:**
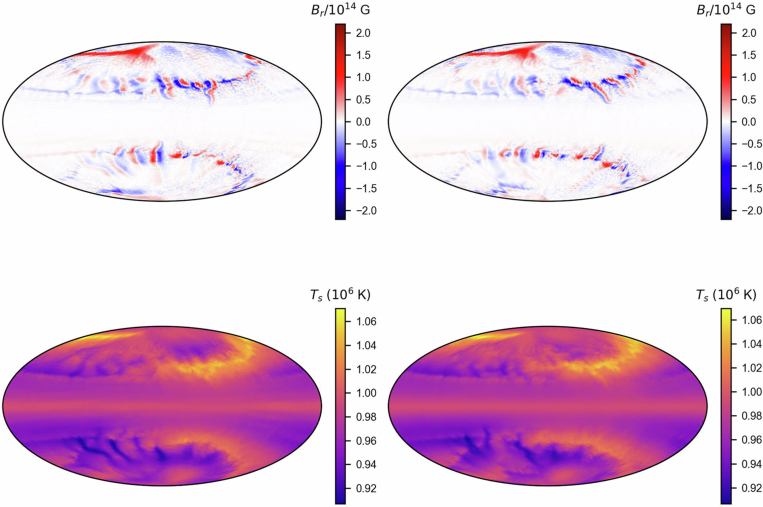
Comparison of simulations with slightly varied initial conditions at 50 kyr. The core temperature is *T*_8_ = 1. Left panel: Initial conditions contain only spherical harmonics up to *ℓ*≤30 from the MHD simulations. Right panel: initial conditions contain up to *ℓ* ≤60. Top panel: surface radial magnetic fields. Bottom panel: surface temperature maps.

In the transition from MHD to eMHD we preserve the angular structures up to *ℓ* = 30, where *ℓ* is the spherical harmonic degree, and downsample the radial part to take into account the differences in simulation set-up such as different geometric aspect ratios and presence of crust in eMHD simulations; full details can be found in Methods and Extended Data Figure 1. We also run a separate shorter simulation keeping angular structure up to *ℓ* ≤ 60, which evolves similarly to our basic one (see Extended Data Figure 2 for comparison).

**Fig. 1:**
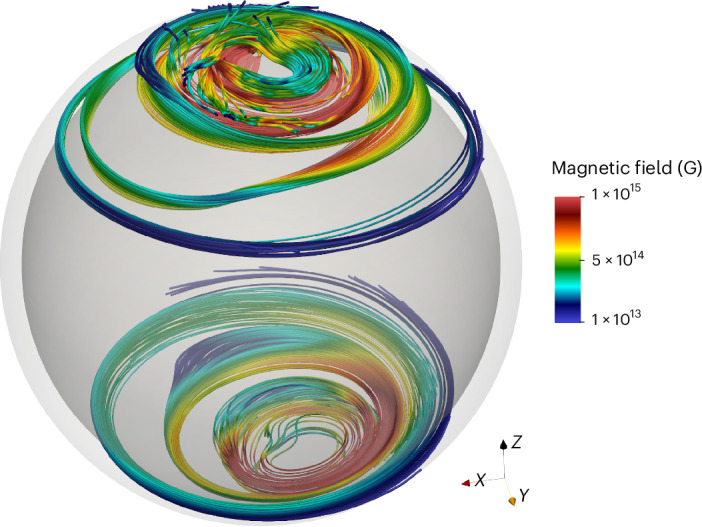
Magnetic-field lines inside the NS crust at the beginning of our NS magnetothermal simulation, that is, at *t* = 0. Starting state for PARODY simulations.

The magnetic field is predominantly toroidal and reaches values up to 3 × 10^15^ G inside the volume, but the field at the outer boundary is much weaker. Assuming a scenario in which the core magnetic field is expelled to a crust-confined configuration, we extract the magnetic field in the top 10% of the simulation volume and adapt it to our code to model crust-confined NS magnetothermal evolution (Methods). Figure 1 shows the initial configuration of the magnetic field inside the NS crust.

We then use the PARODY code to integrate the coupled magnetic induction and thermal diffusion equations for 1 Myr before analysing the NS magnetic characteristics (Methods).

**Fig. 2:**
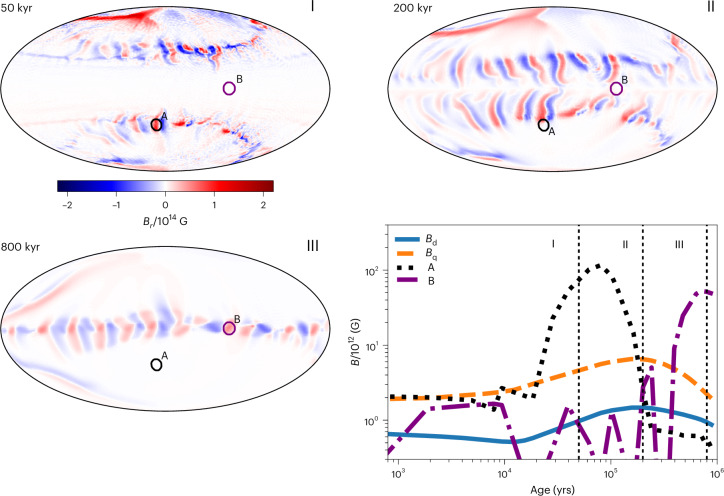
Evolution of surface radial magnetic field. Three maps in Hammer projection showing the surface radial magnetic fields at ages 10 kyr, 200 kyr and 800 kyr. The bottom right panel shows the evolution of the dipole (blue solid line) and quadrupole (orange dashed line) components as well as strengths of radial magnetic fields at the centres of regions A and B marked in each map. The results are obtained using the PARODY code.

Figure 2 shows the dipolar and quadrupolar poloidal magnetic-field intensities, which are the only components that could contribute substantially to electromagnetic spin-down. The surface dipolar magnetic field increases by a factor of only three during the first million years, reaching a maximum value of 1.5 × 10^12^ G, and the quadrupole component remains similarly small, with a maximum of around 6 × 10^12^ G. These values are two to three orders of magnitude smaller than the internal magnetic-field strength in the crust.

**Fig. 3:**
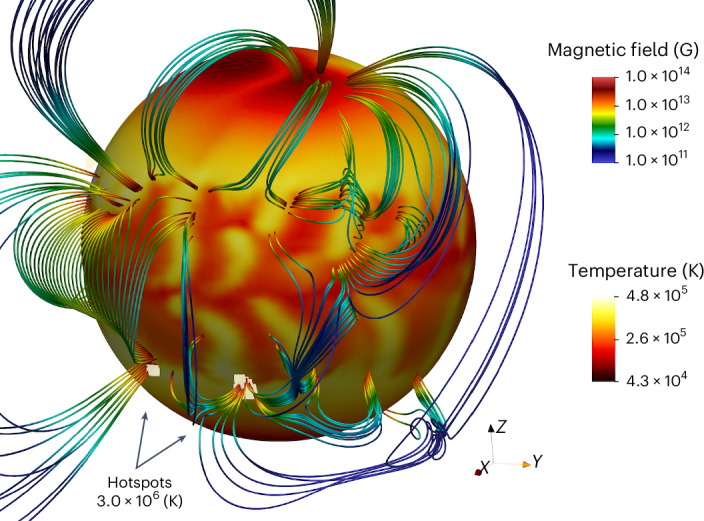
Surface temperature distribution and external magnetic-field structure at age 200 kyr. Magnetospheric hotspots are shown with bright yellow colour. Simulations are performed with the PARODY code.

Figure 3 shows a complex surface magnetic-field topology featuring individual arches elongated in the north–south direction. The local field strength at the footpoints of these arches on the NS surface reaches 10^14^ G, 100 times stronger than the surface dipolar magnetic field. Surface small-scale magnetic fields remain dominant at all times from the beginning of the evolution until 1 Myr later (Fig. 2). Our numerical simulation therefore successfully reproduces two crucial properties of low-field magnetars: (1) weak dipolar magnetic field and (2) presence of very strong (50–100 times stronger) small-scale magnetic fields, similar to those found in SGR 0418+5729^14^ and Swift J1882.3-1606^15^.

## Surface temperatures and hotspots

The X-ray observations of low-field magnetars are consistent with thermal emission from isolated hotspots with sizes of ≤1 km (ref. ^34^) and black-body temperatures reaching *T*_bb_ = 0.12–0.6 keV. The bulk NS emission is not detected with typical upper limits less than 10^31^ erg s^−1^. SGR 0418+5729 has a pulsed fraction of 62 ± 10% in the [0.3–1.2] keV range^34^. CXOU J164710.2-455216 and Swift J1822.3-1606 have quiescent pulsed fractions 80 ± 3% and 38 ± 3% in the [0.5, 10] keV range respectively^35^. The upper limit on the bulk thermal emission indicates that low-field magnetars are at least approximately 200 kyr old because the bulk X-ray emission drops below 10^31^ erg s^−1^ after 200 kyr (ref. ^36^) for strongly magnetized NSs (with internal field strengths of approximately 10^15^ G).

**Extended Data Fig. 3:**
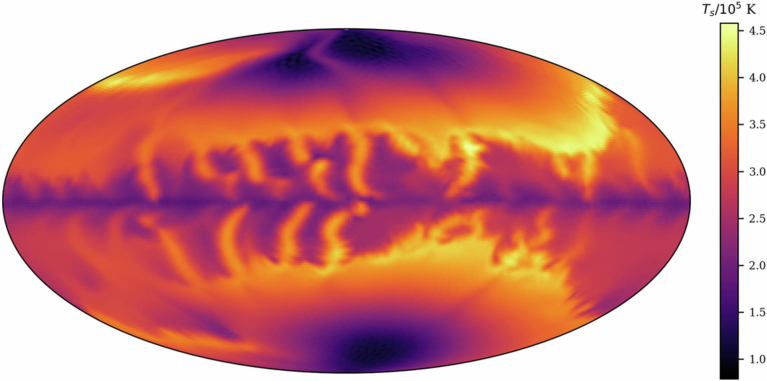
The surface temperature after 200 kyr of evolution. NS core temperature is 10^6^ K. No magnetospheric hot spots are shown.

Strong magnetic fields can create large variations in surface temperature, as illustrated in Fig. 3 (see also Extended Data Figure 3). We see variations of an order of magnitude between the hottest (*T* ≈ 4.8 × 10^5^ K) and coldest (*T* ≈ 4.3 × 10^4^ K) regions. These variations could cause up to 20% pulsed fraction but would stay undetectable because of the low bulk X-ray luminosity, 10^31^ erg s^−1^, and small effective *T*_bb_ = 0.028 keV.

We suggest that the observational properties of low-field magnetars can be explained by magnetospheric heating at the footpoints of small-scale magnetic arches visible in Fig. 3. Magnetospheric currents flow along the field lines and heat the surface, forming hotspots. The size of individual footpoints is a fraction of a kilometre, thus emission generated from these footpoints would have properties of emission seen from low-field magnetars, that is, very high temperature and small emission area.

**Extended Data Fig. 4:**
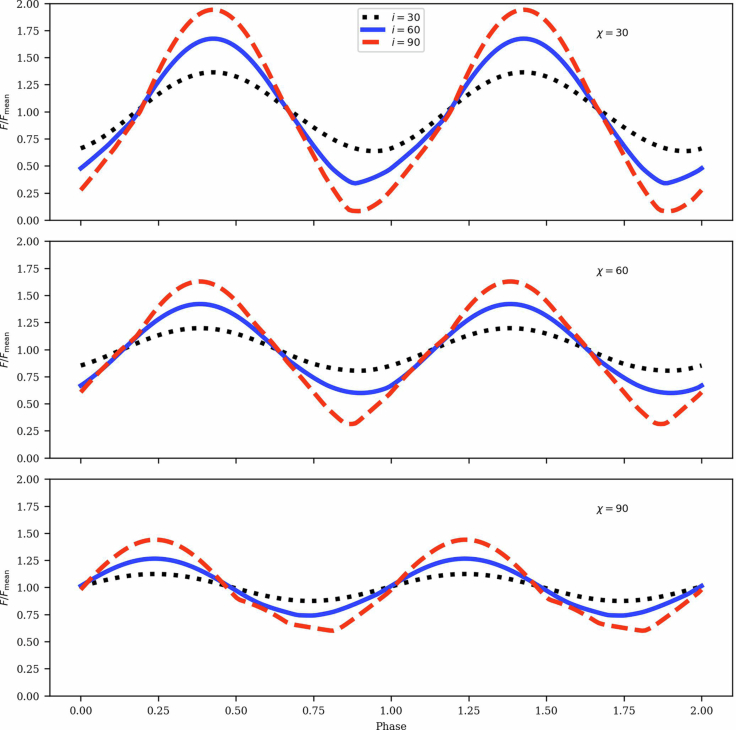
Soft X-ray lightcurves for the surface thermal map combined with the magnetospheric hot spots. Each panel corresponds to a different obliquity angle *χ*. The three curves correspond to different inclination angles: black dotted lines are for *i* = 30^∘^, blue solid lines are for *i* = 60^∘^, and red dashed lines are for *i* = 90^∘^.

**Extended Data Fig. 5:**
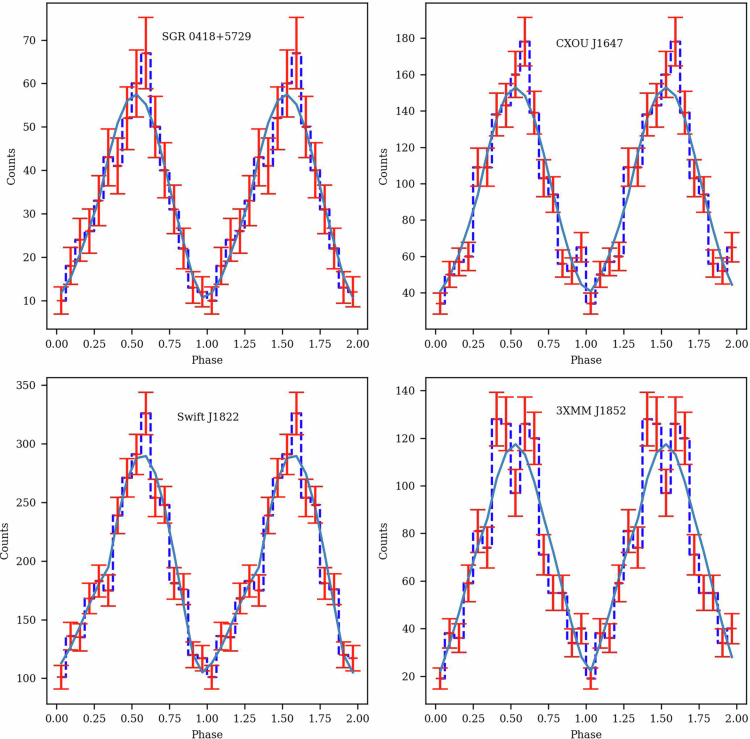
Observed soft X-ray lightcurves for low-field magnetars in the quiescent state compared with model. Data are presented as mean values ± SD. It is assumed that emission is produced by hot spots formed at the places with radial magnetic field exceeding 7 × 10^13^ G. The observed lightcurves are produced by considering energy range of 0.3-2 keV. The best fits (solid blue line) correspond to parameters shown in Table 1.

Models of the magnetosphere generally assume the force-free condition **J** ∝ **∇** × **B** = *μ***B** (ref. ^37^). This means that the strongest radial currents in the magnetosphere near the NS surface coincide with the strongest surface radial magnetic fields. These radial currents heat the surface, producing magnetospheric hotspots. Here we assume that only footpoints with radial magnetic field ∣*B*_*r*_∣ > 7 × 10^13^ G are heated. Under this assumption, it is possible to form up to ten independent hotspots (Fig. 3), which, if heated to 3 × 10^6^ K, produce luminosity 2 × 10^32^ erg s^−1^ and an emission area with radius ≈ 0.9 km. The lightcurve is sine-like with a pulsed fraction reaching 92% for a favourable orientation even without beaming, in agreement with X-ray observations of low-field magnetars (Extended Data Figs. 4 and 5; see also Table 1. Increasing the critical ∣*B*_*r*_∣ leads to fewer hotspots with smaller areas, while decreasing the critical ∣*B*_*r*_∣ results in a larger heated area. If the X-ray thermal emission is indeed generated close to the footpoints of these arches, the arches themselves provide natural sites where Compton scattering occurs and absorption features are formed^14^.

**Table 1:** Rotational orientation for low-field magnetars

Low-field magnetar	*χ* (rad)	*i* (rad)	Δ*Φ* (rad)	C-stat
SGR 0418+5729	0.6984	1.264	5.616	6.8
CXOU J164710.2-455216	0.7518	1.085	5.555	29.9
Swift J1822.3-1606	0.0519	0.636	5.625	16.5
3XMM J185246.6+003317	1.093	1.637	5.330	34.7

*χ* is the obliquity angle, *i* is the inclination between rotational axis and direction towards the observer and Δ*Φ* is initial phase. It is assumed that X-ray lightcurves are produced by hotspots. The last column corresponds to the statistical quality of the fit measured in terms of C-statistics.

## Magnetar bursts

**Fig. 4:**
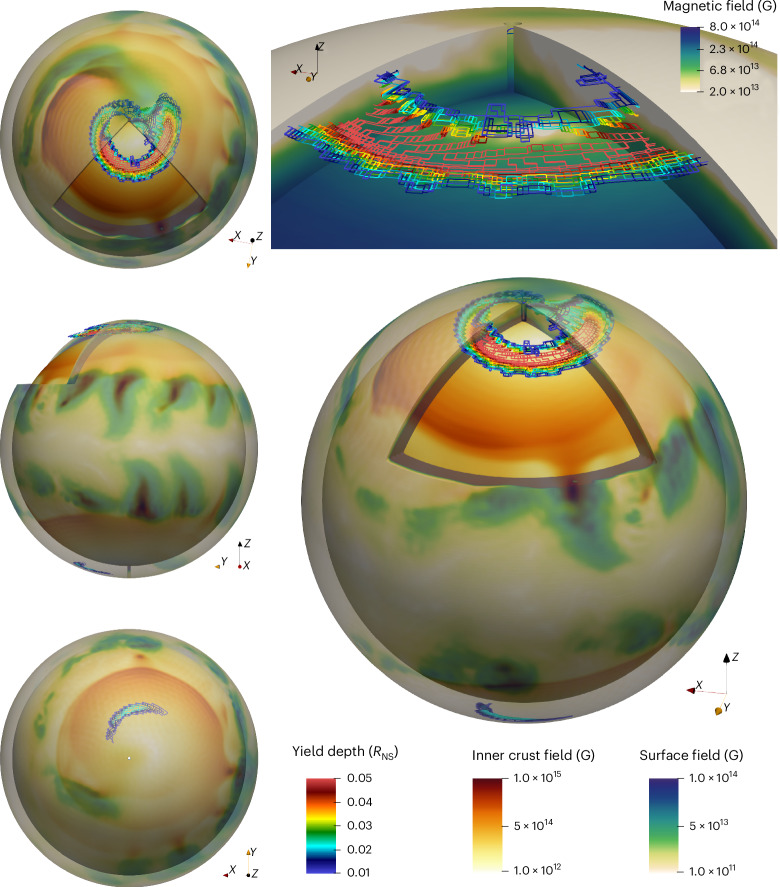
Crust yielding regions. We show surface and inner crust (up to *r* = 0.9 *R*_NS_) magnetic field developed by 200 kyr. Crust yielding regions are indicated by feature edges, colour-coded by depth. Top right: a close-up view of the polar region, with the NS crust colour-coded by magnetic-field intensity.

To assess whether this magnetic-field configuration can power the X-ray activity characteristic of magnetars, we examine the magnetic stresses inside the crust. Bursts and outbursts of magnetars are indeed thought to be caused by crust failure or plastic deformation due to the magnetic stresses^8,38,39^. We apply the Lander–Gourgouliatos model^39^ and compare the crustal magnetic stresses with the von Mises criterion for crust yielding (Methods). To obtain a conservative estimate, the crust is assumed to have completely relaxed only after 2 kyr. Figure 4 shows the average depth of crust failure regions developed at the age of 200 kyr. All the failing regions are located close to the original north and south magnetic poles, coinciding with the regions of strongest magnetic field generated by the proto-NS dynamo. The crust failure regions are much larger in the northern than in the southern hemisphere, due to the properties of the initial magnetic field. This is very different from earlier simulations with simple dipolar initial conditions^40^, in which the crust failure occurred around the original magnetic equator.

The electromagnetic energy that can potentially be released in such a crustal failure is^8^

1 $${E}_{{\rm{out}}}=2\times 1{0}^{39}\,{\rm{erg}}{\left(\frac{l}{1\,{\rm{km}}}\right)}^{2}{\left(\frac{| B| }{2\times 1{0}^{14}\,{\rm{G}}}\right)}^{2},$$

where *l* ≈ 1 km is the typical angular extent of the failing region and ∣*B*∣ ≈ 2 × 10^14^ G as extracted from simulations (Fig. 4). This value is actually well above the typical burst energy of approximately 10^37^ erg of two low-field magnetars: SGR J0418+5729^9^ and CXOU J164710.2-455216^41^. Our modelling provides an upper limit on the extent of crust failure because it maps all the regions that could fail by a certain age.

## Spin periods

**Fig. 5:**
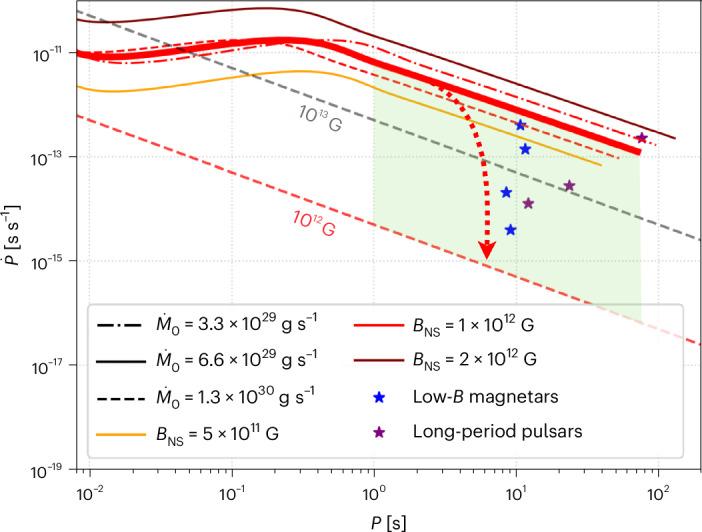
Time evolution of *P* and $\dot{\boldsymbol{P}}$. We show evolution until 10 Myr with small variations of the initial mass accretion rate $\dot{M}$ and dipolar magnetic field *B*_NS_. The dashed straight lines represent the constant-dipolar-magnetic-field lines calculated from the magnetic-dipole spin-down formula for 10^12^ G and 10^13^ G. The green area covers the zone in which the magnetar could end up if the accretion disk is partially depleted for the fiducial parameters *B*_NS_ = 10^12^ G, *M*_d,0_ = 0.01 *M*_⊙_. The red dotted arrow indicates how the $P\dot{P}$ evolution would behave if the disk were completely depleted. Star symbols correspond to observed low-*B* magnetars and long-period pulsars.

By electromagnetic braking alone, NSs with dipolar fields of a few times 10^12^ G cannot reach the spin periods of 8–11 s typical for low-field magnetars on a timescale of 1 Myr. However, it is essential to also take accretion into account, since the Tayler–Spruit dynamo can only develop if the proto-NS accretes fallback material, and this accretion will continue even after the NS is formed. Using the formalism of Ronchi et al.^42^ to model torques from the fallback disk, we naturally obtained periods of 8–11 s after 170 kyr for NSs with a dipolar magnetic field similar to that in our simulations (Fig. 5). More details of these calculations are summarized in Methods.

**Extended Data Fig. 6:**
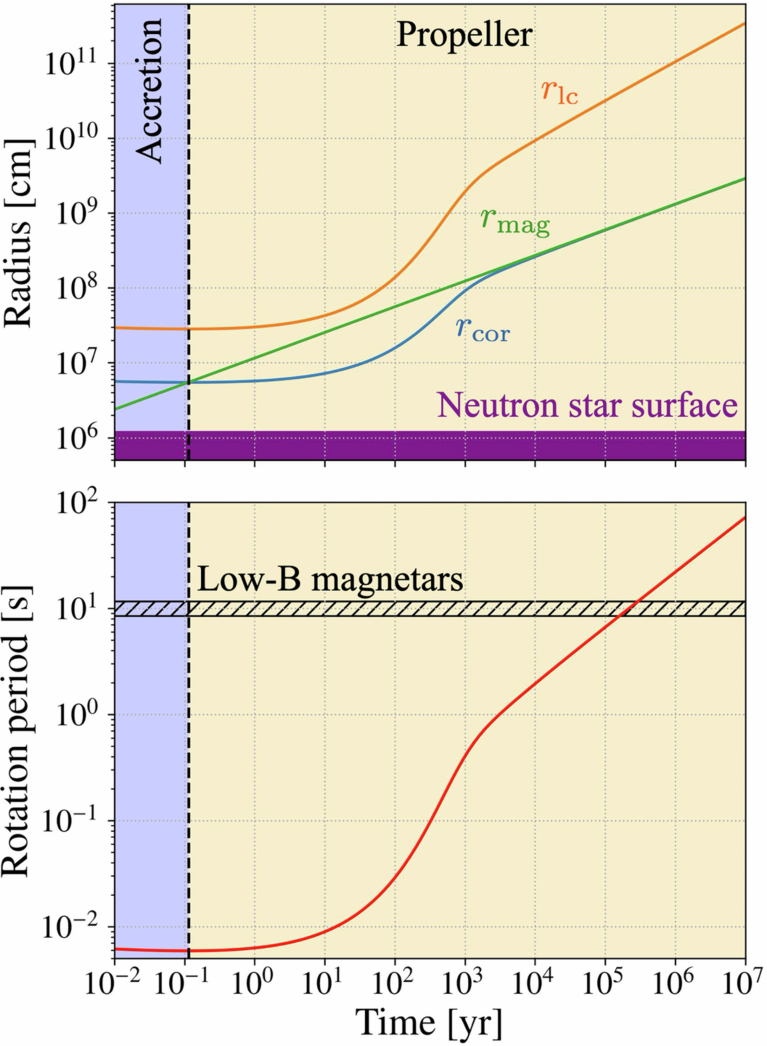
Time evolution of the characteristic radii and the NS rotation period. Top panel: characteristic radii; bottom panel: NS rotation period. We fix magnetic field at value *B*_NS_ = 10^12^ G, *M*_d,0_ =0.01 *M*_⋅_, and an initial rotation period of 10 ms. The NS is spun up during the accretion regime (blue region) and strongly spun down in the propeller phase (orange region). The hatched region in the bottom figure represents the range of rotation periods observed in low-field magnetars. The figure is produced by solving eq. (51).

Most of the spin-down occurs during the propeller stage when the NS decelerates due to the interaction of its magnetosphere with the fallback disk (Extended Data Fig. 6). After 200 kyr, this propeller phase has spun the NS down to a rotation period of *P* = 8.5 s and a period derivative $\dot{P}=8.5\times 1{0}^{-13}$ s s^−1^. According to the standard magnetic dipole spin-down formula, the inferred surface magnetic dipole should then be *B*_dip_ ≈ 3.8 × 10^13^ G, which overestimates the true surface magnetic dipole in our simulation by a factor of approximately 40. This inferred value of *B*_dip_ is comparable to that of Swift J1822.3-1606 (1.4 × 10^13^ G) and below the upper limit measured for CXOU J164710.2-455216 (<6.6 × 10^13^ G) as well as 3XMM J185246.6+003317 (<4 × 10^13^ G).

The apparent magnetic field estimated using the instantaneous period and period derivative might be smaller if the disk is partially depleted and provides less torque. Depending on the exact amount of material left in the disk, the period derivative $\dot{P}$ could range from approximately 10^−15^ (electromagnetic spin-down only) to approximately 10^−12^ (non-depleted disk; green area in Fig. 5). All low-field magnetars with a measured period derivative fall within this area.

## Impact and future work

Previous magnetothermal simulations have considered idealized, large-scale magnetic fields^13^. Some of these simulations can be made more similar to low-field magnetars by assuming a magnetar-strength dipolar magnetic field which is then dissipated by an increased crust resistivity^11^. Moreover, these simulations remain highly axially symmetric because of the symmetries of the initial conditions. Although some previous studies have considered more complicated field structures, as expected from proto-NS evolution^29,30^, our study directly implements the field from a self-consistent dynamo simulation. The crucial properties of our new magnetic-field configuration are that this field is predominantly toroidal and is initially localized near the polar regions of the crust. As a consequence, we find that crustal fractures are most likely to occur in these regions.

In comparison with the model suggested by Rea et al.^11^, with an initial dipolar field as strong as 1.5 × 10^14^ G, the dipolar magnetic field stays very low, approximately 10^12^ G, and does not decay substantially in our simulations.

The following mechanisms have been suggested to be responsible for formation of magnetar magnetic fields: (1) fossil fields^43,44^, (2) convective dynamo^7,17^, (3) dynamo due to MRI^20^ and (4) Tayler–Spruit dynamo^23^. There are also more exotic mechanisms such as the chiral magnetic instability in proto-NSs^45^ leading to the formation of magnetic fields with sub-millimetre to centimetre scales.

Despite its simplicity, the fossil-field hypothesis is among the most uncertain ones since the magnetic-field configuration would be subject to multiple instabilities during the proto-NS stage. If the fossil field does survive the proto-NS stage, the strengths of poloidal and toroidal components should be roughly the same^43^, which would point towards classical magnetars where the dipolar component is large and comparable to the strength of toroidal fields.

Raynaud et al.^17^ show that the convective dynamo can lead to classical magnetar formation if the proto-NS rotates very rapidly (spin period < 10 ms). Rapidly rotating proto-NSs are expected to be rare, and most proto-NSs should have longer spin periods. In the slow-rotation case, the convective dynamo works in the turbulent regime and would be expected to form mostly small-scale magnetic fields. It was suggested^46^ that, due to an inverse Hall cascade, small-scale fields decay while large-scale fields grow. Other papers^27,47^ were inspired by this turbulent dynamo and tried to test the inverse Hall cascade. The initial conditions were created by injecting energy for 10 ≤ *ℓ* ≤ 20. The outcome of these simulations was central compact objects because the average field was designed to be smaller than the one required to cause crustal yielding. Moreover, only a very limited inverse cascade was found. These papers^27,47^ did not reproduce any actual dynamo simulations.

In a recent paper, Dehman et al.^30^ performed a simulation of magnetothermal evolution starting with an initial magnetic configuration similar to one produced by MRI^48^. They focused on energy spectra and did not try to precisely reproduce the spatial distribution of electric currents, similarly to refs. ^27,47^. Note also that, unlike the Tayler–Spruit dynamo, the MRI develops when the proto-NS has a radius *R* ≈ 40 km, which is notably larger than that of a typical NS, which forced ref. ^30^ to rather roughly mimic this contraction as no simulation has ever been made of the relaxation of MRI-generated magnetic fields. Similar to our research, they^30^ found no substantial growth of the dipolar component, and most of the total magnetic energy was stored in small-scale magnetic fields. They interpreted observational properties of their objects as similar to those of central compact objects. It is unknown if their configuration leads to the formation of surface patches with strong radial magnetic field or to crust yielding. In their scenario, there is no need to consider a fallback disk as the rapid rotation originates from the progenitor; these NSs should thus have much shorter spin periods, which indeed makes them more similar to central compact objects.

The following aspects of the Tayler–Spruit dynamo scenario (considering proto-NSs with rotation periods longer than 6 ms) make it especially suitable for the formation of low-*B* magnetars: (1) formation of strong toroidal magnetic fields necessary for magnetar activity, (2) weak dipole and quadrupole at the surface, which naturally explain low-*B* magnetars, (3) presence of small-scale fields, which form patches of very strong radial magnetic field as a result of the Hall evolution, and (4) presence of a fallback disk, which allows the NS to gain a long spin period. Note that the Tayler–Spruit dynamo may also generate a stronger magnetic dipole and lead to the formation of a classical magnetar for rotation periods shorter than 6 ms (P.B., J.G., R.R. & A. Reboul-Salze, manuscript under review). Note that such a rotation period is close to the minimum initial proto-NS period derived from the kinetic energy of supernova remnants associated with magnetars^24^.

Our results also suggest an important connection between low-field magnetars and recently discovered long-period radio pulsars, such as PSR J0901-4046^49^. If the NS continues to operate in the propeller phase, it will ultimately reach periods comparable to 75 s by 10 Myr (Fig. 5). The external magnetic-field configuration remains complex, with large open field-line curvature near the NS surface facilitating radio pulsar operation. Thus, pulsar radio emission could occur if the disk is depleted.

Mahlmann et al.^50^ performed numerical simulations for X-ray outbursts with energies up to 10^43^ erg produced by a twisted magnetar magnetosphere. In our simulations, we see the development of individual magnetic arcs and the evolution of their footpoints. Thus, our results can be used as the initial magnetic field for future relativistic magnetosphere simulations. An additional open question is how magnetospheric currents flowing along small-scale arches will be sustained by strong-field quantum electrodynamic reactions and plasma dynamics in the magnetosphere.

Our work opens new perspectives for testing extreme dynamos operating in proto-NSs. We suggest that different dynamos leave their unique imprint on magnetic-field configurations, thus allowing us to identify different magnetic amplification processes using the magnetothermal properties of young isolated NSs. While we suggest that the formation of low-field magnetars is linked to the Tayler–Spruit dynamo, the formation of classical magnetars as well as the internal structure of their magnetic fields remains an open question.

## Methods

### Simulation of the proto-NS dynamo

We simulate a proto-NS with a mass of 1.4 *M*_⊙_ and a radius *R*_NS_ = 12 km. Its interior is modelled as a stably stratified fluid enclosed between two spherical shells. To control the differential rotation, we impose constant rotation frequencies on both shells (spherical Taylor–Couette configuration), with the outer shell rotating faster than the inner shell to be consistent with the fallback formation scenario. We solve the Boussinesq MHD equations by using the pseudospectral code MagIC^51,52^. In this code, the different lengths *r*, the time *t*, the temperature *T* and the magnetic field *B* are scaled as follows:

2 $$r\to rd,\qquad t\to\left({d}^{\,2}/\nu\right)t,\qquad T\to ({T}_{\mathrm{o}}-{T}_{\mathrm{i}})T,\qquad B\to \sqrt{4\uppi \rho \eta {\varOmega }_{\mathrm{o}}}B,$$

with the gap between the two spheres *d* = *r*_o_ − *r*_i_ = 9 km, the kinematic viscosity *ν* = 3.5 × 10^9^ cm^2^ s^−1^, the temperatures of the outer and inner spheres *T*_o_ and *T*_i_ respectively, the constant density *ρ* = 4.1 × 10^14^ g cm^−3^, the resistivity *η* = 3.5 × 10^9^ cm^2^ s^−1^ and *Ω*_o_ = 628 rad s^−1^. So, the dimensionless equations solved using MagIC read

3 $$\boldsymbol{\nabla }\cdot \mathbf{v}=0,$$

4 $$\boldsymbol{\nabla }\cdot \mathbf{B}=0,$$

5 $$\frac{{\mathrm{D}}\mathbf{v}}{{\mathrm{D}}t}+\frac{2}{E}\,{\mathbf{e}}_{z}\times \mathbf{v}=-\boldsymbol{\nabla }\varPi +\frac{\mathrm{Ra}}{\mathrm{Pr}}\,T{\mathbf{e}}_{r}+\frac{1}{E\,{\mathrm{Pm}}}\,(\boldsymbol{\nabla }\times \mathbf{B})\times \mathbf{B}+\Delta \mathbf{v},$$

6 $$\frac{{\mathrm{D}}T}{{\mathrm{D}}t}=\frac{1}{\mathrm{Pr}}\,\Delta T,$$

7 $$\frac{\partial \mathbf{B}}{\partial t}=\boldsymbol{\nabla }\times (\mathbf{u}\times \mathbf{B})+\frac{1}{\mathrm{Pm}}\,\Delta \mathbf{B},$$

where **v** and **B** are the velocity and magnetic field. The potential *Π* includes all gradient forces and functions as a Lagrange multiplier to ensure the solenoidality of the velocity field (equation (4)). D/D*t* ≡ ∂/∂*t* + **v**·**∇** is the Lagrangian derivative. *E*, Ra, Pr and Pm are dimensionless numbers, which depend on the fluid properties. The Ekman number *E* is defined as the ratio of the rotation period to the viscous timescale,

8 $$E=\frac{\nu }{{d}^{2}{\varOmega }_{\mathrm{o}}}=1{0}^{-5}.$$

The thermal and magnetic Prandtl numbers are defined by

9 $${\mathrm{Pr}}=\frac{\nu }{\kappa }=0.1\qquad {\rm{and}}\qquad {\mathrm{Pm}}=\frac{\nu }{\eta }=1,$$

where *κ* = 3.5 × 10^10^ cm^2^ s^−1^ is the thermal diffusivity. Finally, the Rayleigh number Ra measures the ratio between the timescales of thermal transport by diffusion to the thermal transport by convection,

10 $${\mathrm{Ra}}={\left(\frac{N}{{\varOmega }_{0}}\right)}^{2}\frac{\mathrm{Pr}}{{E}^{\,2}},$$

where

11 $$N\equiv \sqrt{-\frac{{g}_{0}}{\rho }\left({\left.\frac{\partial \rho }{\partial S}\right\vert }_{P,{Y}_{e}}\frac{{\mathrm{d}}S}{{\mathrm{d}}r}+{\left.\frac{\partial \rho }{\partial {Y}_{e}}\right\vert }_{P,S}\frac{{\mathrm{d}}{Y}_{e}}{{\mathrm{d}}r}\right)}=68.2\,{{\rm{s}}}^{-1}$$

is the Brunt–Väisälä frequency. The gravitational acceleration is assumed to be purely radial, **g** = g_0_*r*/*r*_0_**e**_*r*_. *Y*_*e*_ and *S* are the electron fraction and the entropy, respectively.

We apply no-slip, electrically insulating and fixed-temperature boundary conditions on both shells. The spin-up torque exerted at the outer boundary by the choice of a fixed angular velocity mimics in an approximate way the effect of fallback accretion. The resolution used is (*n*_*r*_, *n*_*θ*_, *n*_*ϕ*_) = (257, 256, 512), where *n*_*r*_, *n*_*θ*_ and *n*_*ϕ*_ are numbers of grid points in the radial, latitudinal and longitudinal direction. For more information on the numerical methods, see the supplementary materials of ref. ^23^.

### Conversion between MagIC and PARODY codes

There are substantial differences between MagIC and PARODY codes. In particular, in MagIC the radial part of the potentials is expanded using Chebyshev polynomials, while in PARODY the radial part is represented using finite differences. While PARODY covers only the NS crust, the MagIC simulations include a core. Therefore, we downsample the result of MagIC simulations in the radial direction.

The poloidal–toroidal decompositions and thus the magnetic potentials are defined differently in the MagIC and PARODY codes. Specifically,

12 $$\mathbf{B}=\boldsymbol{\nabla }\times \boldsymbol{\nabla }\times\left({b}_{{\rm{pol}}}^{{\rm{M}}}{\mathbf{e}}_{r}\right)+\boldsymbol{\nabla }\times\left({b}_{{\rm{tor}}}^{{\rm{M}}}{\mathbf{e}}_{r}\right),$$

13 $$\mathbf{B}=\boldsymbol{\nabla }\times \boldsymbol{\nabla }\times\left({b}_{{\rm{pol}}}^{{\rm{P}}}r{\mathbf{e}}_{r}\right)+\boldsymbol{\nabla }\times\left({b}_{{\rm{tor}}}^{{\rm{P}}}r{\mathbf{e}}_{r}\right),$$

where the superscript M/P refers to MagIC/PARODY, respectively.

Moreover, the codes use different normalization factors *C*_*lm*_ for the spherical harmonics ${Y}_{l}^{\,m}(\theta ,\phi )$. The spherical harmonics are normalized in the PARODY code as

14 $${C}_{lm}^{{\rm{P}}}=\sqrt{\left(2-{\delta }_{m,0}\right)\left(2l+1\right)\frac{\left(l-m\right)!}{\left(l+m\right)!}},$$

while the normalization in the MagIC code reads

15 $${C}_{lm}^{{\rm{M}}}=\frac{1}{1+{\delta }_{m,0}}\sqrt{\frac{(2l+1)}{4\uppi }\frac{(l-m)!}{(l+m)!}},$$

where *δ*_*m*,0_ is the Kronecker delta.

Thus, for the radial magnetic field, we have

16 $${B}_{r}=\frac{l(l+1)}{{r}_{{\rm{m}}}^{2}}\,{b}_{{\rm{pol}}}^{lm,{\rm{M}}}({r}_{{\rm{m}}})\,{C}_{lm}^{{\rm{M}}}\,{Y}_{l}^{\,m}(\theta ,\phi )=\frac{l(l+1)}{{r}_{{\rm{p}}}}\,{b}_{{\rm{pol}}}^{lm,{\rm{P}}}({r}_{{\rm{p}}})\,{C}_{lm}^{{\rm{P}}}\,{Y}_{l}^{\,m}(\theta ,\phi ).$$

Making this comparison for each (*l*, *m*) separately we thus obtain

17 $${b}_{{\rm{pol}}}^{lm,{\rm{P}}}\left({r}_{{\rm{p}}}\right)={b}_{{\rm{pol}}}^{lm,{\rm{M}}}\left({r}_{{\rm{m}}}\right)\,\frac{{r}_{{\rm{p}}}}{{r}_{{\rm{m}}}^{2}}\frac{{C}_{lm}^{{\rm{M}}}}{{C}_{lm}^{{\rm{P}}}}.$$

Expanding and simplifying this expression we obtain two different equations for axisymmetric and non-axisymmetric poloidal potentials,

18 $${b}_{{\rm{pol}}}^{l0,{\rm{P}}}({r}_{{\rm{p}}})=\frac{{b}_{{\rm{pol}}}^{l0,{\rm{M}}}({r}_{{\rm{m}}})}{\sqrt{4\uppi }}\frac{{r}_{{\rm{p}}}}{{r}_{{\rm{m}}}^{2}}\quad {\rm{for}}\quad m=0,$$

19 $${b}_{{\rm{pol}}}^{lm,{\rm{P}}}({r}_{{\rm{p}}})=\frac{{b}_{{\rm{pol}}}^{lm,{\rm{M}}}\left({r}_{{\rm{m}}}\right)}{\sqrt{2\uppi }}\quad {\rm{for}}\quad m\ne 0.$$

Similarly, we can proceed with the *θ* component of the magnetic field computed using only the toroidal potential

20 $${B}_{\theta }=\frac{{C}_{lm}^{{\rm{M}}}}{{r}_{{\rm{m}}}\sin \theta }\,{b}_{{\rm{tor}}}^{lm,{\rm{M}}}\left({r}_{{\rm{m}}}\right)\,\frac{\partial {Y}_{l}^{\,m}(\theta ,\phi )}{\partial \phi }=\frac{{C}_{lm}^{{\rm{P}}}}{\sin \theta }\,{b}_{{\rm{tor}}}^{lm,{\rm{P}}}\left({r}_{{\rm{p}}}\right)\,\frac{\partial {Y}_{l}^{\,m}(\theta ,\phi )}{\partial \phi }.$$

Thus, the normalization is

21 $${b}_{{\rm{tor}}}^{lm,{\rm{P}}}\left({r}_{{\rm{p}}}\right)={b}_{{\rm{tor}}}^{lm,{\rm{M}}}\left({r}_{{\rm{m}}}\right)\,\frac{{C}_{lm}^{{\rm{M}}}}{{C}_{lm}^{{\rm{P}}}}\frac{1}{{r}_{{\rm{m}}}},$$

which simplifies to

22 $${b}_{{\rm{tor}}}^{l0,{\rm{P}}}\left({r}_{{\rm{p}}}\right)=\frac{{b}_{{\rm{tor}}}^{l0,{\rm{M}}}\left({r}_{{\rm{m}}}\right)}{\sqrt{4\uppi }}\frac{1}{{r}_{{\rm{m}}}}\quad {\rm{for}}\quad m=0,$$

23 $${b}_{{\rm{tor}}}^{lm,{\rm{P}}}\left({r}_{{\rm{p}}}\right)=\frac{{b}_{{\rm{tor}}}^{lm,{\rm{M}}}\left({r}_{{\rm{m}}}\right)}{\sqrt{2\uppi }}\frac{1}{{r}_{{\rm{m}}}}\quad {\rm{for}}\quad m\ne 0.$$

In this work, we preserve the angular structure obtained in dynamo simulations at the surface and in the middle of the crust up to *ℓ* = 30, which corresponds to surface structures of approximately 1 km. In addition, we ran shorter simulations where we preserve the angular structure up to *ℓ* = 30. We compare the results in Extended Data Figure 1 and show that they are very similar to our basic simulation.

### Crust-confined magnetic-field configurations

In addition to the technical details in the previous section, the proto-NS dynamo set-up and the magnetothermal crust evolution set-up differ in their geometry, having geometric aspect ratios *χ*_pNS_ = 0.25 and *χ*_NS_ = 0.9, respectively. Thus, to create a magnetic-field configuration that is similar to proto-NS results but is also crust confined, we should extract only the top 10% of the proto-NS simulation.

Our approach for importing the results of the dynamo simulations is to require all components of the magnetic field to coincide between MHD and eMHD simulations at certain points within the crust. We consider the poloidal and toroidal potentials for each individual spherical harmonic, and require both these potentials to exactly coincide with our numerical fits at the following points: *r*_1_ = 0.93 and *r*_2_ = 0.96. We require our fit for the poloidal potential to coincide at the surface. We also require our poloidal and toroidal potentials to satisfy the potential boundary condition at the surface and the ‘no-currents’ boundary condition at the core–crust interface.

Similarly to recent work^53^, we represent the radial part of the poloidal and toroidal potentials as a polynomial expansion

24 $${b}_{lm}(r)=\frac{{a}_{0}+{a}_{1}r+{a}_{2}{r}^{2}+{a}_{3}{r}^{2}+{a}_{4}{r}^{\,4}}{r}.$$

Overall, all conditions for the radial part of the poloidal potential can be written as

25 $$\begin{array}{cll}{b}_{p}(1)&=&{\beta }_{p}(1.0),\\ {b}_{p}(0.96)&=&{\beta }_{p}(0.96),\\ {b}_{p}(0.93)&=&{\beta }_{p}(0.93),\\ {b}_{p}({r}_{c})&=&0,\\ \frac{\partial {b}_{p}}{\partial r}(1)+\frac{(l+1)}{r}{b}_{p}(1)&=&0.\end{array}$$

Here *β*_*p*_(*r*) are coefficients of the spectral expansion for poloidal magnetic field extracted from the proto-NS MagIC simulations. These conditions are individually satisfied for each *l* and *m*, and translate into the following system of linear equations:

26 $$\begin{array}{lcl}{a}_{0}+{a}_{1}+{a}_{2}+{a}_{3}+{a}_{4}&=&{\beta }_{p}(1.0),\\\left({a}_{0}+{a}_{1}{r}_{1}+{a}_{2}{r}_{1}^{2}+{a}_{3}{r}_{1}^{\,3}+{a}_{4}{r}_{1}^{\,4}\right)/{r}_{1}&=&{\beta }_{p}({r}_{1}),\\\left({a}_{0}+{a}_{1}{r}_{2}+{a}_{2}{r}_{2}^{2}+{a}_{3}{r}_{2}^{\,3}+{a}_{4}{r}_{2}^{\,4}\right)/{r}_{2}&=&{\beta }_{p}({r}_{2}),\\ {a}_{0}+{a}_{1}{r}_{c}+{a}_{2}{r}_{c}^{2}+{a}_{3}{r}_{c}^{\,3}+{a}_{4}{r}_{c}^{\,4}&=&0,\\ {a}_{0}l+(l+1){a}_{1}+(l+2){a}_{2}+(l+3){a}_{3}+(l+4){a}_{4}&=&0.\end{array}$$

For the toroidal potential we use the following conditions:

27 $$\begin{array}{lcl}{b}_{t}(1)&=&0,\\ {b}_{t}(0.96)&=&{\beta }_{t}(0.96),\\ {b}_{t}(0.93)&=&{\beta }_{t}(0.93),\\ \partial \left[r{b}_{t}({r}_{c})\right]/\partial r&=&0.\end{array}$$

Similarly, *β*_*t*_(*r*) here are the coefficients of the spectral expansion for the toroidal magnetic field extracted from the proto-NS simulations. These conditions then translate into the linear system

28 $$\begin{array}{lcc}{a}_{0}+{a}_{1}+{a}_{2}+{a}_{3}&=&0,\\\left({a}_{0}+{a}_{1}{r}_{1}+{a}_{2}{r}_{1}^{2}+{a}_{3}{r}_{1}^{3}\right)/{r}_{1}&=&{\beta }_{t}({r}_{1}),\\\left({a}_{0}+{a}_{1}{r}_{2}+{a}_{2}{r}_{2}^{2}+{a}_{3}{r}_{2}^{3}\right)/{r}_{2}&=&{\beta }_{t}({r}_{2}),\\ {a}_{1}+2{a}_{2}{r}_{c}+3{a}_{3}{r}_{c}^{2}=0.\end{array}$$

### Simulation of NS magnetothermal evolution

Code PARODY^54–56^ was modified to solve the magnetothermal evolution of NSs. The test cases (benchmarks) for the code are available in the literature^57^. The pseudospectral code PARODY was modified to solve the following system of dimensionless partial differential equations for **B** and *T*:

29 $$\frac{\partial \boldsymbol{B}}{\partial t}={\rm{Ha}}\,\boldsymbol{\nabla }\times \left[\frac{1}{{\mu }^{3}}\mathbf{B}\times (\boldsymbol{\nabla }\times \mathbf{B})\right]-\boldsymbol{\nabla }\times \left[\frac{1}{{\mu }^{2}}\boldsymbol{\nabla }\times \mathbf{B}\right]+{\rm{Se}}\,\nabla \left[\frac{1}{\mu }\right]\times \boldsymbol{\nabla }{T}^{2},$$

30 $$\frac{{\mu }^{2}}{{\rm{Ro}}}\,\frac{\partial {T}^{2}}{\partial t}=\boldsymbol{\nabla }\cdot \left[{\mu }^{2}\hat{\chi }\cdot \boldsymbol{\nabla }{T}^{2}\right]+\frac{{\rm{Pe}}}{{\rm{Se}}}\,\frac{| \boldsymbol{\nabla }\times \mathbf{B}{| }^{2}}{{\mu }^{2}}+{\rm{Pe}}\,\mu \left[\boldsymbol{\nabla }\times \mathbf{B}\right]\cdot \boldsymbol{\nabla }\left[\frac{{T}^{2}}{{\mu }^{2}}\right],$$

where the first equation is the magnetic induction equation and the second is the thermal diffusion equation. The terms on the right-hand side of the first equation correspond to the Hall effect, Ohmic decay and the Biermann battery effect. The terms on the right-hand side of the second equation correspond to anisotropic thermal diffusion, Ohmic heating and entropy carried by electrons. The derivation of the above equations is summarized in ref. ^57^. The same code was also used to compute the evolution of off-centred dipole configurations^53^.

To ensure the solenoidality of **B**, we write the magnetic field as a sum of poloidal and toroidal parts,

31 $$\mathbf{B}=\boldsymbol{\nabla }\times \boldsymbol{\nabla }\times ({b}_{{\rm{pol}}}\mathbf{r})+\boldsymbol{\nabla }\times ({b}_{{\rm{tor}}}\mathbf{r}).$$

The scalar potentials *b*_pol_ and *b*_tor_ are expanded in spherical harmonics.

The electron chemical potential varies within the crust as

32 $$\mu (r)={\mu }_{0}{\left[1+\frac{(1-r/{R}_{{\rm{NS}}})}{0.0463}\right]}^{4/3}.$$

The electrical resistivity varies within the crust, but is not sensitive to temperature in our simulations. Our chosen parameters roughly mimic resistivity caused by impurity with parameter *Q*_imp_ ≈ 40. In a real NS the resistivity is larger during the first 10 kyr, which means that the role of Hall evolution is slightly smaller during this time. In our simulations, we are interested in longer timescales where the Hall evolution dominates. The tensor $\hat{\chi }$ describing the anisotropy of the heat transport is written as

33 $$\hat{\chi }=\frac{{\delta }_{ij}+{\rm{Ha}}\,{B}_{i}{B}_{j}/{\mu }^{2}-{\rm{Ha}}\,{\epsilon }_{ijk}{B}_{k}/\mu }{1+{{\rm{Ha}}}^{2}\,| \mathbf{B}{| }^{2}/{\mu }^{2}},$$

where *δ*_*ij*_ is the Kronecker symbol and *ϵ*_*ijk*_ is the Levi-Civita symbol.

The dimensionless Hall (Ha), Seebeck (Se), Péclet (Pe) and Roberts (Ro) parameters depend on the chosen scales for the magnetic field and temperature, which we take to be *B*_0_ = 10^14^ G and *T*_0_ = 1.0 × 10^8^ K. The Hall number is defined by

34 $${\rm{Ha}}=c{\tau }_{0}\frac{e{B}_{0}}{{\mu }_{0}}\approx 49.1,$$

where *e* is the electron charge, *c* is the speed of light, *τ*_0_ = 9.9 × 10^19^ s is the electron scattering relaxation time^58^ and *μ*_0_ = 2.9 × 10^−5^ erg is the electron chemical potential at the top of the crust. The Seebeck number is defined by

35 $${\rm{Se}}=2{\uppi }^{3}{k}_{{\rm{B}}}^{2}{T}_{0}^{2}{n}_{0}e\frac{c{\tau }_{0}}{{\mu }_{0}{B}_{0}}\approx 0.052,$$

where *k*_B_ is the Boltzmann constant and *n*_0_ = 2.603 × 10^34^ cm^−3^ is the electron number density at the top of the crust. Finally, the Péclet and Roberts numbers are

36 $${\rm{Pe}}=\frac{3}{4\uppi }\frac{{B}_{0}}{e{n}_{0}c{\tau }_{0}}\approx 6.44\times 1{0}^{-5}$$

and

37 $${\rm{Ro}}=\frac{3}{4{\uppi }^{3}}\frac{{\mu }_{0}^{2}}{{k}_{{\rm{B}}}{T}_{0}}\frac{1}{{c}^{2}{\tau }_{0}^{2}}\frac{1}{{e}^{2}{n}_{0}}\approx 3{,}580.$$

We model the core as a perfect conductor, which implies the following inner boundary conditions at *r* = 0.9:

38 $${b}_{{\rm{pol}}}=0\qquad {\rm{and}}\qquad \frac{{\mathrm{d}}(r{b}_{{\rm{tor}}})}{{\mathrm{d}}r}=0.$$

We model the region outside the NS as a vacuum, which implies the following outer boundary conditions at *r* = 1:

39 $$\frac{{\mathrm{d}}{b}_{\,\text{pol}}^{lm}}{{\mathrm{d}}r}+\frac{l+1}{r}{b}_{\text{pol}\,}^{lm}=0\qquad {\rm{and}}\qquad {b}_{{\rm{tor}}}=0,$$

where ${b}_{\,\text{pol}\,}^{lm}$ is the coefficient of degree *l* and order *m* in the spherical harmonic expansion of the poloidal potential *b*_pol_.

The temperature is fixed to its initial value at the core–crust boundary (see more details about modelling cooling at the end of the section). The outer boundary condition for the temperature is

40 $$-{\mu }^{2}\mathbf{r}\cdot \hat{\chi }\cdot \boldsymbol{\nabla }\left({T}^{2}\right)=\frac{1}{5}\frac{{R}_{{\rm{NS}}}}{c{\tau }_{0}}\,{\rm{Se}}\,{\rm{Pe}}\,{\left({T}_{\mathrm{s}}/{T}_{0}\right)}^{4},$$

where the (dimensional) surface temperature *T*_s_ is related to the crustal temperature *T*_b_ as

41 $${\left[\frac{{T}_{\mathrm{s}}}{1{0}^{6}\,{\rm{K}}}\right]}^{2}=\left[\frac{{T}_{\mathrm{b}}}{1{0}^{8}\,{\rm{K}}}\right],$$

using a simplified relation^59^.

The numerical resolution is *n*_*r*_ = 96 grid points in the radial direction and spherical harmonic degrees up to *l*_max_ = 128.

To take into account the NS cooling, we restart calculations at 200 kyr, changing the core temperature to 10^6^ K. We run calculations for 1 kyr to allow the simulation to relax, that is, crust temperatures stop evolving on short timescales, creating a stable surface thermal pattern. The timescale of 1 kyr is estimated numerically and is related to assumptions of how we model the thermal capacity of the crust.

### Properties of thermal emission

We use the open-source code Magpies to model X-ray thermal lightcurves. We show these results in Extended Data Figure 4. The maximum pulsed fraction reaches 93% for the most favourable orientation of the rotational axis with respect to the original dipole axis.

Similarly to ref. ^13^, we try to fit the soft-X-ray lightcurve in the range 0.3–2 keV. We show the results in Extended Data Figure 5. We summarize the obliquity angle as well as inclination angles in Table 1. While SGR 0418+5729 and Swift J1822.3-1606 are fitted relatively well, the two remaining magnetars have more features in the lightcurves.

### Crust failure

We use here a model developed in ref. ^39^ based on earlier work in ref. ^38^. Essentially, we use the von Mises criterion for crust yielding, following equation (14) of ref. ^39^:

42 $${\tau }_{{\rm{el}}}\le \frac{1}{4\uppi }\sqrt{\frac{1}{3}{\mathbf{B}}_{0}^{4}+\frac{1}{3}{\mathbf{B}}^{4}+\frac{1}{3}{\mathbf{B}}_{0}^{2}{\mathbf{B}}^{2}-{\left(\mathbf{B}\cdot {\mathbf{B}}_{0}\right)}^{2}}.$$

Here **B**_0_ is the relaxed (initial) state of the magnetic field, which we assume to coincide with our first simulation snapshot at 2 kyr. *τ*_el_ is the scalar yield stress. **B** is computed at 200 kyr. We compute the critical strain following the procedure in ref. ^39^ with a correction (S. Lander, personal communication)

43 $$\tilde{\rho }=99.6{\left(1-\frac{{R}_{{\rm{cc}}}}{{R}_{{\rm{nd}}}}\right)}^{2}{(1-{\mathcal{R}})}^{2}+0.004,$$

where ${\mathcal{R}}$ is computed as

44 $${\mathcal{R}}=\frac{r-{R}_{{\rm{cc}}}}{{R}_{{\rm{nd}}}-{R}_{{\rm{cc}}}},$$

*R*_cc_ = 0.9 is the location of the crust–core interface and *R*_nd_ = 1 is the location of the neutron drip point. Thus, our critical strain varies from approximately 8 × 10^26^ g cm^−1^ s^−2^ close to the neutron drip boundary to 4.6 × 10^29^ g cm^−1^ s^−2^ at the core–crust boundary. Following our normalization, the stress caused by Lorentz forces (right-hand side of equation (42)) is multiplied by a numerical factor, (10^14^ G)^2^. This von Mises criterion is written assuming that failure occurs in the form of shearing motion^39^.

### Accretion-driven spin-down

To explain the NS spin-down to the regime of low-field magnetars, we invoke the propeller mechanism^60^ due to the interaction between the NS magnetic field and the remaining fallback disk. The evolution of the NS fallback depends on the three different radii: (1) the light-cylinder radius, (2) the magnetospheric radius and (3) the corotation radius, which are defined by the respective expressions

45 $${r}_{{\rm{lc}}}=\frac{c}{{\varOmega }_{{\rm{NS}}}},$$

46 $${r}_{{\rm{mag}}}={\mu }^{4/7}{(G{M}_{{\rm{NS}}})}^{-1/7}{\dot{M}}^{-2/7},$$

47 $${r}_{{\rm{cor}}}={\left(\frac{G{M}_{{\rm{NS}}}}{{\varOmega }_{{\rm{NS}}}^{2}}\right)}^{1/3}.$$

*M*_NS_ and *Ω*_NS_ are the NS mass and rotation rate; *μ* = *B*_NS_*R*_NS_^3^ is its magnetic dipole moment. $\dot{M}$ is the accretion rate. Strictly speaking, $\dot{M}$ is the material loss rate from the accretion disk. In the propeller regime this quantity remains positive even though the material is not accreted onto the NS.

If the disk penetrates the magnetosphere (*r*_lc_ > *r*_mag_), it can either spin up the NS by accreting matter if *r*_cor_ > *r*_mag_, or spin down the NS in a propeller phase if *r*_cor_ < *r*_mag_. In this propeller phase, the magnetic field accelerates the inner disk to super-Keplerian speeds, which produces a centrifugal outflow. Angular momentum is therefore transported from the NS toward the disk, which can efficiently spin down the NS.

The modelling of the NS fallback evolution we use is strongly inspired by ref. ^61^ except for the mass accretion rate, which reads^42^

48 $$\dot{M}(t)={\dot{M}}_{0}{\left(1+\frac{t}{{t}_{\nu }}\right)}^{-1.2},$$

where *t*_*ν*_ ≈ 30 s is the viscous timescale and ${\dot{M}}_{0}={M}_{{\rm{d}},0}/{t}_{\nu }\approx 6.5\times 1{0}^{29}$ g s^−1^ is the initial accretion rate, and *M*_d,0_ = 0.01 *M*_⊙_ is the initial fallback disk mass. The torques exerted on the NS by the accretion disk are given by

49 $${N}_{{\rm{acc}}}=\left\{\begin{array}{ll}\left(1-{\left(\frac{{r}_{{\rm{mag}}}}{{r}_{{\rm{cor}}}}\right)}^{3/2}\right)\sqrt{G{M}_{{\rm{NS}}}{R}_{{\rm{NS}}}{\dot{M}}^{2}}\quad &{\rm{if}}\,{r}_{{\rm{mag}}} > {R}_{{\rm{NS}}},\\ \left(1-{\left(\frac{{\varOmega }_{{\rm{NS}}}}{{\varOmega }_{{\rm{K}}}}\right)}^{3/2}\right)\sqrt{G{M}_{{\rm{NS}}}{R}_{{\rm{NS}}}{\dot{M}}^{2}}\quad &{\rm{if}}\,{r}_{{\rm{mag}}} < {R}_{{\rm{NS}}},\end{array}\right.$$

where ${\varOmega }_{{\rm{K}}}=\sqrt{G{M}_{{\rm{NS}}}/{R}_{{\rm{NS}}}^{3}}$ is the Keplerian angular velocity. The dipole spins the NS down as follows:

50 $${N}_{{\rm{dip}}}=-\frac{2}{3}\frac{{\mu }^{2}{\varOmega }_{{\rm{NS}}}^{3}}{{c}^{3}}{\left(\frac{{r}_{{\rm{lc}}}}{{r}_{{\rm{mag}}}}\right)}^{3}.$$

Therefore, the NS angular velocity evolves as

51 $${I}_{{\rm{NS}}}{\dot{\varOmega }}_{{\rm{NS}}}={N}_{{\rm{acc}}}+{N}_{{\rm{dip}}},$$

where *I*_NS_ = 1.45 × 10^45^ g cm^2^ is the NS moment of inertia. Extended Data Figure 6 shows the time series of the characteristic radii and NS rotation period that result from the solution of equation (51) for *B*_NS_ = 10^12^ G, *M*_d,0_ = 0.01 *M*_⊙_ and an initial rotation period of 10 ms. We clearly find that the NS is strongly spun down during the propeller phase and reaches the period range of the observed low-field magnetars at approximately 170 kyr. This timescale varies up to approximately 550 kyr for *B*_NS_ = 5 × 10^11^ G.

## Data Availability

We have made data underlying all main figures publicly available in the Zenodo repository: 10.5281/zenodo.14335239^62^.

## Code availability

For the proto-NS simulation, we used the MagIC code (commit 2266201a5), which is open source at https://github.com/magic-sph/magic. The magnetar spin-down was calculated with the GRB code (commit 84788793), also publicly available at https://github.com/rraynaud/GRBs. We used the Magpies code, publicly available at https://github.com/ignotur/magpies (commit a1fde77). The PARODY code is available only upon request in its original version.
